# Cat Predation on Bats in an Urban Landscape of Eastern Europe: Twelve Years of Rescue-Centre Records

**DOI:** 10.3390/ani16213536

**Published:** 2026-11-09

**Authors:** Anton Vlaschenko, Maryna Yerofeieva, Alona Shulenko, Valeriia Bohodist, Kateryna Zemliana, Andrii Kulynych, Veronika Putiatina, Alona Prylutska

**Affiliations:** 1 Ukrainian Bat Rehabilitation Center, NGO “Ukrainian Independent Ecology Institute”, 61001 Kharkiv, Ukraine; 2 Educational and Research Bat Biology Laboratory, Institute of Natural Sciences, H.S. Skovoroda Kharkiv National Pedagogical University, 61168 Kharkiv, Ukraine; 3 Leibniz Institute for Zoo and Wildlife Research, 10315 Berlin, Germany; 4 National Scientific Center “Institute of Experimental and Clinical Veterinary Medicine”, 61023 Kharkiv, Ukraine; 5 School of Biology, V.N. Karazin Kharkiv National University, 61077 Kharkiv, Ukraine; 6 Finnish Museum of Natural History, University of Helsinki, 00014 Helsinki, Finland; 7 Falz-Fein Biosphere Reserve “Askania Nova” of the National Academy of Agrarian Sciences of Ukraine, 01010 Kyiv, Ukraine

**Keywords:** Chiroptera, *Felis catus*, urban ecology, wildlife rehabilitation, *Eptesicus serotinus*, *Nyctalus noctula*, ecological trap, lyssavirus, One Health, Ukraine

## Abstract

Predation by domestic cats is a recognised but poorly quantified threat to bats, and Eastern Europe is almost absent from the evidence. We analysed twelve years (2013–2024) of records of the Ukrainian Bat Rehabilitation Centre, 41,910 bats of 15 species from 258 settlements, to estimate how often a cat was implicated in a find. Among the 4770 bats received in finds of four individuals or fewer, 1.59% (95% CI 1.27–1.99) were cat-related, with no trend over the years. The odds of a cat-related case were four to five times higher for *Eptesicus serotinus* and *Vespertilio murinus* than for *Nyctalus noctula*, and in the activity season seven to ten times higher for these two species and for *Pipistrellus kuhlii*. The male excess among *E. serotinus* victims matched the sex composition of the bats received, and age had no effect. Cat attacks were fatal for 54% of the bats concerned, but not more often than injuries of other origin. In one building in Kharkiv, by contrast, cats established inside killed 14.8% of the 1099 bats collected in a single winter, and predation fell to the background level after the cats were killed and the crevices sealed. Cat predation on urban bats thus operates in two regimes, a dispersed background at a stable low rate and localised roost-scale events governed by access, which call for different measures.

## Simple Summary

Domestic cats are among the most numerous predators in cities, and bats are among the animals they catch. How often this happens has rarely been measured. We examined twelve years of records of a bat rescue network in Ukraine, covering 41,910 bats from 258 settlements, and identified 258 bats that had been caught by a cat. Two situations emerged. About one of every sixty bats brought in singly or in small numbers had been caught by a cat, and this rate did not change over twelve years. In one building in Kharkiv, cats living inside killed at least 163 bats in a single winter; predation there fell to the low level seen elsewhere after the cats were killed and the entrances were sealed. Species that roost in buildings were caught most often, usually outdoors or in the yards of private houses. The common noctule, whose roosts normally lie beyond a cat’s reach, was rarely caught except inside that one building. The species caught most often, the common serotine, is also the main carrier of a bat lyssavirus in Europe, and three-quarters of the bats caught by cats reach people alive. This contact therefore matters for conservation and for public health.

## 1. Introduction

Anthropogenic modification of the environment is a principal driver of contemporary change in the distribution and abundance of terrestrial vertebrates [1,2]. Its effects extend beyond habitat loss to the redistribution of species [3]. Geographic ranges contract or expand, and population densities are restructured within them, so that local abundance increasingly reflects the availability of anthropogenic resources rather than the properties of natural habitats [4,5]. Urbanisation is the most pronounced expression of this process. Urban assemblages have no analogue in natural or pristine habitats. Taxa that would seldom co-occur are brought into regular contact, at encounter rates governed by human infrastructure rather than by the processes that ordinarily regulate the populations involved.

Predator–prey interactions are among those most strongly affected by urbanisation. In natural systems, predator abundance is constrained by prey availability, and predation pressure declines as prey become scarce. This regulation does not operate for human-subsidised predators. Their populations are sustained by anthropogenic food and shelter, independently of the abundance of wild prey, and they continue to hunt in the absence of nutritional requirement [6,7]. Predation pressure on urban prey may therefore exceed that of ecologically comparable wild predators by several orders of magnitude, and it is not relaxed as prey decline. Prey that a predator takes only incidentally may nonetheless suffer demographically significant mortality.

The domestic cat *Felis catus* Linnaeus, 1758 is the most extensively documented predator of this type. Densities of owned and unowned cats in European settlements greatly exceed those of wild carnivores of comparable body mass: 324–463 cats per km^2^ in residential areas of a British town [8], and 26 cats per km^2^ for unowned free-ranging cats alone in rural Denmark, where their national population is estimated at 89,000 [9]. Predatory behaviour is retained irrespective of nutritional state, and prey acquisition varies markedly among individuals, a minority accounting for a disproportionate share of recorded kills [7,10]. National estimates of the resulting mortality reach tens of millions of prey items annually in Great Britain [11] and billions in the United States [12]. Such estimates derive largely from prey returned to households. They therefore under-represent prey that is nocturnal, difficult to retrieve or infrequently transported, categories that include bats [11,13].

Bats are common members of the urban fauna of Europe, and in some cities become locally abundant during autumn swarming and hibernation [14,15,16]. Several species roost in crevices and cavities of buildings and forage in association with artificial lighting [17,18]; in parts of Eastern Europe, large hibernating aggregations form within multi-storey buildings that function as analogues of rock faces [19,20]. Cats have been reported to prey upon at least 86 bat species, approximately 7% of extant species, with Vespertilionidae over-represented among recorded victims [13]. Rescue-centre data indicate a higher apparent incidence than dietary studies: in peninsular Italy, cat predation was the most frequent cause of admission of adult bats, accounting for 28.7% of records [21]. This figure is likely conservative. Cat DNA was recovered from approximately two-thirds of injured bats submitted by rehabilitators in the United Kingdom, most recorded only as exhibiting wing tears of undetermined origin [22,23]. Where several species share a roost, cats do not take them in proportion to their abundance: at a cave in Puerto Rico, the species killed most often were not the most numerous [24].

The frequency with which urban bat populations encounter cats, and the species involved, nonetheless remain largely unquantified. This gap is relevant to conservation and to public health alike. Predation establishes direct contact between a wild reservoir host of lyssaviruses and a domestic carnivore that moves freely between wild and domestic environments [25], and transmission across the cat–bat interface, including of lyssaviruses against which current vaccines are unlikely to protect, has been documented in Europe [26,27,28,29,30,31]. Urban environments therefore concentrate contact between human-commensal predators and wild reservoir hosts, and represent an interface at which the conservation and One Health dimensions of cat predation converge.

Eastern Europe is poorly represented within this body of evidence, although the relevant conditions are present. In Ukraine, EBLV-1a was identified in *Vespertilio murinus* Linnaeus, 1758 as early as 1987, and lyssavirus infection has since been recorded in a domestic cat that had been in contact with a bat, and in a bat recovered from a cat [32]. In the only global synthesis of bat species subject to cat predation, Ukraine is represented by a single study [13], which documented sustained predation by free-roaming cats upon a hibernating aggregation of *Nyctalus noctula* (Schreber, 1774) in one building in Kharkiv, where more than 150 carcasses were recovered during a single winter [19]. A further national record, not included in that synthesis, concerns repeated captures of bats by an individual domestic cat in the south of the country [33]. Both refer to single localities. The Ukrainian Bat Rehabilitation Centre (Bats Ukraine) has received bats and documented the circumstances of each admission since the early 2010s [34], and its network of regional partners and volunteers became fully operational in 2021 [35]. These records have not previously been analysed with respect to cat predation.

Here we analyse twelve complete years of records from this centre. We determine the proportion of the bats received that is attributable to contact with cats, test whether bat species are affected in proportion to their representation among the bats received, and examine the seasonal, sexual and age structure of these cases.

## 2. Materials and Methods

### 2.1. Dataset

Records were collected by the Ukrainian Bat Rehabilitation Centre (UBRC), a rescue network that receives bats from the public throughout Ukraine. Its procedures, species composition and geographical coverage have been described previously [34,35], and the occurrence records are published through GBIF [36,37]. The records originate predominantly from urban and suburban settlements and were generated by members of the public submitting bats to the centre.

Each record refers to a single bat and holds the date, the settlement and address at which it was found, the species, sex, age and morphometric measurements, and the fate of the animal. Three free-text fields hold the circumstances of the find as reported by the finder or noted by staff.

The working datasets cover January 2013 to December 2024 without interruption and comprise 41,910 bats of 15 species from 258 settlements. Coverage of the country is uneven. Kharkiv city alone supplied 60.9% of the bats received. Dnipro, Poltava, Zaporizhzhia and Kamianske cities follow, and with Kharkiv these five settlements account for 83.2% of the material. The rest is thinly spread: 205 of the 258 settlements yielded fewer than ten bats each.

### 2.2. Identification of Cat-Related Cases

The three free-text fields of every record were screened for terms denoting a cat, and every record returned was read and classified by hand. A record was accepted as a cat-related case when a cat was named as the agent of the injury, or when the finder or a member of staff attributed the injuries to a cat. Each case corresponds to one bat. The material comprises 258 cat-related cases in all: 77 in the general dataset, 163 at the Derzhprom building in Kharkiv in the winter of 2014/15 and 18 at the same building in other seasons (Table 1). Every case is listed individually in Table S1, with the original free-text note and its English translation.

### 2.3. Finds: Definition and Size

Bats reach the centre in two ways. Most are found and reported by residents, singly or in small numbers. The rest arrive from rescue operations, in which an entire aggregation trapped inside a building is recovered over one or a few days and brought into care.

A find is defined by address and date: all bats taken from one address on one date constitute a single find. In the general dataset, 3681 finds consisted of a single bat; no cat-related case occurred among the 29,952 bats received in finds of 20 or more individuals.

Percentages are reported throughout at four thresholds on the size of the find: finds of a single bat; finds of four bats or fewer; finds of up to 15 bats, the largest find in which a cat-related case occurred; and all finds. The statistical models described in Section 2.5 are fitted to finds of four bats or fewer. Finds of four bats or fewer follow a single encounter and hold 76 of the 77 cases of the general dataset. Larger finds come mainly from rescue operations and contribute almost no cases: one case among the 32,236 bats received in finds of more than four individuals. The estimated percentage therefore falls as larger finds are admitted. The two classes combined here are not homogeneous: single-bat finds and finds of two to four bats differ in species and seasonal composition (Table S4). Unless stated otherwise, the percentages and contrasts reported for the general dataset in Section 3 refer to finds of four bats or fewer.

### 2.4. The Derzhprom Building Case

One building in Kharkiv, the Derzhprom building, has been described previously as a site of cat predation on a hibernating aggregation of *N. noctula* [19]. Because a single site of this kind would dominate any pooled estimate, it was analysed separately.

The 4904 bats received from the Derzhprom building between January 2013 and December 2024 are treated in two parts. The first is the winter of 2014/15, from September 2014 to March 2015. For these 1099 bats, the cause of death of every individual was determined from the specimen and recorded in a dedicated dataset [19], and 163 were classified as killed by cats. The second is the remaining 3805 bats received in other seasons, among which screening identified 18 cases.

The main building of V.N. Karazin Kharkiv National University, on the same square 200 m from the Derzhprom building and holding a hibernating aggregation of the same species, served as a comparison. Its records form part of the general dataset and were examined separately (Section 3.6).

### 2.5. Statistical Analysis

The analyses are descriptive at the level of the whole material and inferential within species. Percentages are given with Wilson 95% confidence intervals, and proportions were compared with Fisher’s exact test. All analyses were carried out in Python 3.12.3 with pandas 3.0.2, NumPy 2.4.4, SciPy 1.17.1 and statsmodels 0.15.0.

Period, sex, age and outcome were examined within species, because the species differ both in the period at which they reach the centre and in the percentage of bats for which a cat is recorded. Four species had at least 100 individuals among finds of four bats or fewer and were analysed in this way: *N. noctula*, *Eptesicus serotinus* (Schreber, 1774), *Vespertilio murinus* and *Pipistrellus kuhlii* (Kuhl, 1817), 4678 bats and 75 cases in all (Table 1); the remaining eleven species, 92 bats with one case, are reported descriptively. For each contrast, odds ratios were calculated within each species from the corresponding 2 × 2 table, with Wald confidence intervals and a continuity correction of 0.5 where a cell was empty; homogeneity between species was tested with the Breslow–Day–Tarone test, and a stratified Mantel–Haenszel estimate with species as the stratum was obtained. Stratified estimates are reported only where homogeneity was not rejected. These contrasts treat bats as independent; since 22.8% of the bats in finds of four or fewer arrived in finds of two to four individuals, their confidence intervals may be slightly narrower than warranted. No correction was applied for multiple comparisons. The contrasts within species are exploratory, several rest on five or six cases, and the interpretation follows the stratified estimates rather than any single within-species value.

The association between species and the occurrence of a cat-related record was modelled with a binomial generalised linear model (GLM) with a logit link, fitted to the same four species with species as the only predictor and *N. noctula* as the reference level. Odds ratios are given with Wald confidence intervals. Because bats received in the same find are not independent, the model was also fitted by generalised estimating equations with an exchangeable working correlation and the find as the cluster. It was refitted at each of the other three thresholds on the size of the find and to the activity season, April to October, in which all four species are present. Full output is given in Table S2.

The year was divided into five phenological periods following the division used for this bat assemblage in earlier work [16,34,38]: hibernation from November to March, spring migration in April, breeding from May to July, autumn swarming in August and September, and pre-hibernation fat accumulation in October. Calendar seasons were not used, since in this assemblage November and March belong to the hibernation period. Period was treated as a binary contrast, first between the activity season and hibernation and then between the breeding period and the rest of the year. Age compared adults with first-year bats, that is, volant young of the year, and excluded juveniles, non-volant young still dependent on the mother. Bats whose sex or age was not determined were excluded from the corresponding analysis; age was recorded for 66 (88.0%) of the 75 cases; and bats whose month of receipt was not recorded (40 of the 4678) were excluded from the contrasts by period.

The outcome of each bat was taken from the status field of the database together with the free-text fields and assigned to one of five categories: dead when received, died in care, euthanised, released, or not determined.

A fatal outcome comprises the first three categories and was analysed as a binary response against two comparison groups: all other bats received, and only those other bats in whose free-text record an injury was described, identified by a search for terms denoting fractures, wounds, bites, tears, haematomas, bleeding and necrosis. In the cat-related cases of the general dataset, the injuries described in the note were further assigned to five categories, which are not mutually exclusive: fracture of the wing bones; damage to the wing membrane; wounds on the head, trunk or limbs; loss of a body part; and haematoma, swelling or bleeding (Table S1). The place of the find was derived from the free-text fields and assigned to one of six categories: outdoors, on the ground or in the street away from any building; the yard of a private house, that is, the enclosed ground of a single-family dwelling; indoors, meaning the inside of a dwelling or office to which the public has no access; a balcony or window, including a window well or an external ledge; a stairwell or building entrance, that is, the communal circulation space of a block; and a basement or attic, that is, the communal service spaces of a block. For the Derzhprom building, the type of place was taken from the corresponding field of the dedicated dataset. Places are reported descriptively, and no statistical comparison between them is made, for the reasons set out in Note S1.

### 2.6. Use of Generative Artificial Intelligence

During the preparation of this manuscript, the authors used Claude (Anthropic; models Claude Opus 5.5 and Claude Fable 5.1) to edit the structure and language of the text. The study design, the data collection, and the statistical analyses were carried out by the authors. The authors have reviewed and edited the output and take full responsibility for the content of this publication.

## 3. Results

### 3.1. Frequency of Cat-Related Cases

Of the 77 cat-related cases in the general dataset, 75 were finds of a single bat, and 76 were finds of four bats or fewer; no case occurred among the 29,952 bats received in finds of 20 or more individuals (Table 2). The percentage of cat-related cases therefore depended on the threshold applied: it was 2.04% among finds of a single bat, 1.59% (95% CI 1.27–1.99) among finds of four bats or fewer and 0.21% when all finds were included (Table 2). Finds of more than four bats came almost entirely from rescue operations, in which an aggregation trapped inside a building was recovered by the staff of the centre rather than reported by a resident after an encounter.

**Figure 1:**
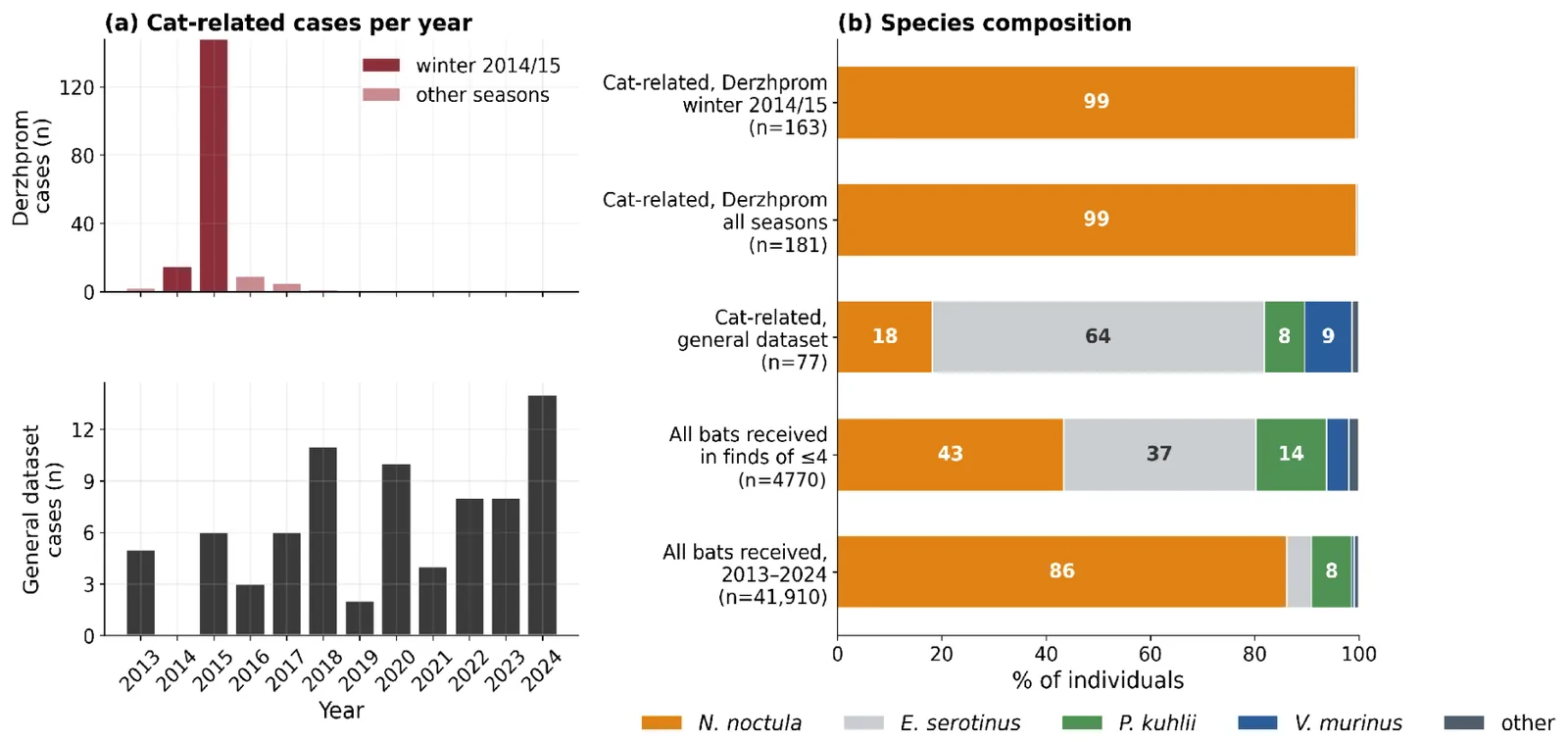
(**a**) Annual number of cat-related cases at the Derzhprom building (**upper** panel) and in the general dataset (**lower** panel). (**b**) Species composition of cat-related cases and of the bats received, from the single winter at one building to the dataset as a whole. The fourth bar gives the composition of the finds of four bats or fewer used in the analysis.

Cases occurred in every year except 2014, and the annual percentage ranged from 0 to 3.12% with no directional trend (odds ratio 1.01 per year, 95% CI 0.94–1.08, *p* = 0.84; Figure 1a). Kharkiv contributed 3590 of the 4761 bats with a recorded settlement (75.4%) and 65 of the 76 cases; the eleven cases outside Kharkiv came from eleven different settlements. The percentage of cat-related cases did not vary with the number of bats received from a settlement (odds ratio 1.22 per tenfold increase, 0.95–1.57, *p* = 0.115; Note S2). It was higher in Kharkiv than in the rest of the country (1.80% against 0.95%, OR = 1.92, *p* = 0.043), but the difference was not significant among finds of a single bat (2.22% against 1.37%, OR = 1.63, *p* = 0.157; Note S3).

At the Derzhprom building, 163 of the 1099 bats collected in the winter of 2014/15 were classified as killed by cats (14.83%, 95% CI 12.87–17.03), twice the twelve-year total of the general dataset (Figure 1a; Section 3.6). *Eptesicus serotinus* accounted for 49 of the 77 cases in the general dataset (63.6%, 95% CI 52.5–73.5), but for 36.8% of the bats received in finds of four bats or fewer, whereas 162 of the 163 cases at Derzhprom were *N. noctula* (Figure 1b).

### 3.2. Cat-Related Cases by Bat Species

**Figure 2:**
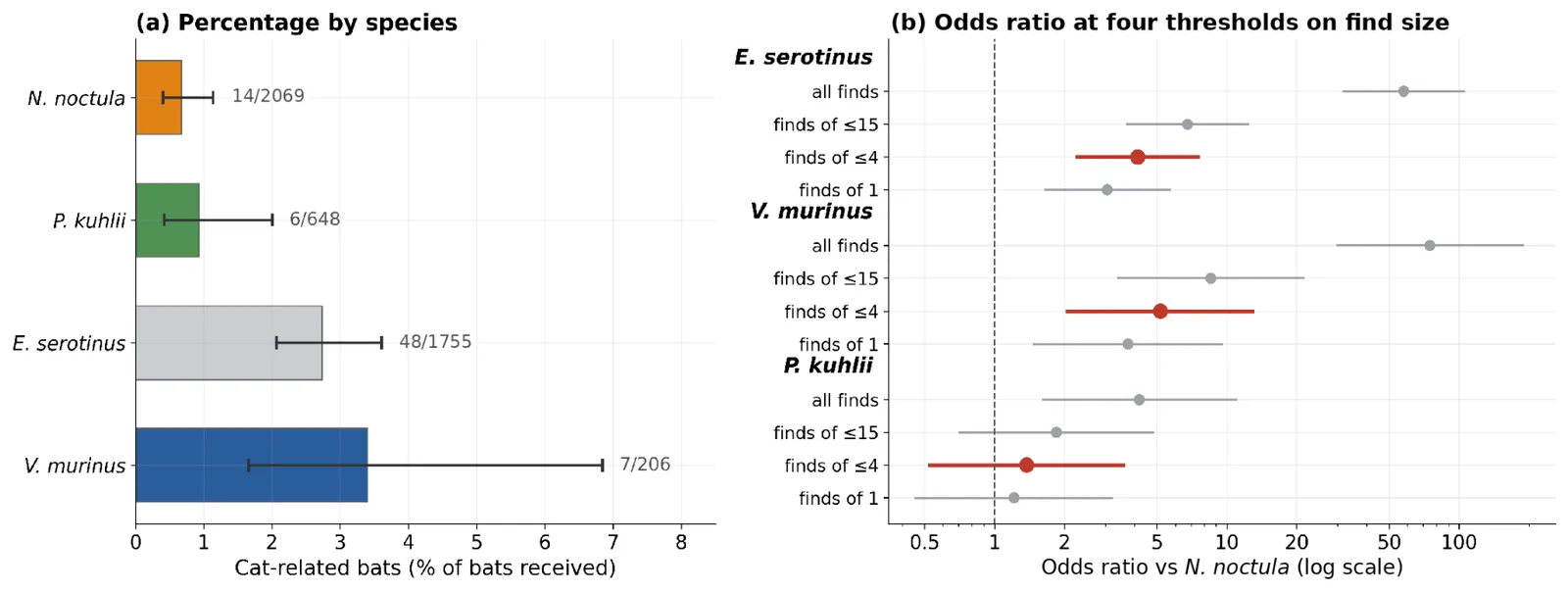
(**a**) Percentage of bats received in finds of four or fewer individuals for which a cat was recorded, by species, with Wilson 95% confidence intervals; the number of cases and the number of bats received are given beside each bar. (**b**) Odds ratios relative to *N. noctula* at four thresholds on the size of the find, on a logarithmic scale. The threshold used in the analysis, finds of four bats or fewer, is shown in red. The other three thresholds are shown in grey; horizontal lines are 95% confidence intervals, and the dashed line marks an odds ratio of 1.

Among bats received in finds of four or fewer individuals, the percentage of cat-related cases differed between the four species (Table 3, Figure 2a). It was highest in *E. serotinus* (2.74%) and *V. murinus* (3.40%), in both of which the odds were four to five times those in *N. noctula* (0.68%); in *P. kuhlii* (0.93%) the odds did not differ from those in *N. noctula*. The model with species as the only predictor was significant as a whole (Table S2). The direction of the differences held at every threshold on the size of the find, and *E. serotinus* and *V. murinus* differed significantly from *N. noctula* at all of them; the odds ratios rose as larger finds were admitted, because those finds added almost no cases and consisted mainly of *N. noctula*, the reference species (Figure 2b, Note S3).

The species differed in the phenological composition of the bats received: hibernation accounted for 79.5% of *N. noctula* and 75.1% of *P. kuhlii*, against 56.8% of *E. serotinus* and 41.7% of *V. murinus* (Table S3a). Among the 1450 bats received from April to October, the activity season in which all four species are present, the odds ratios relative to *N. noctula* were 9.70 for *E. serotinus* (95% CI 2.32–40.64, *p* = 0.002), 9.23 for *V. murinus* (1.77–48.21, *p* = 0.008) and 6.83 for *P. kuhlii* (1.31–35.59, *p* = 0.022), on 45 cat-related cases (Table S2).

Eleven further species were represented by 92 individuals in total, with one cat-related case in *Hypsugo savii* (Bonaparte, 1837); these species were not modelled.

### 3.3. Phenological Period, Sex and Age Within Species

**Figure 3:**
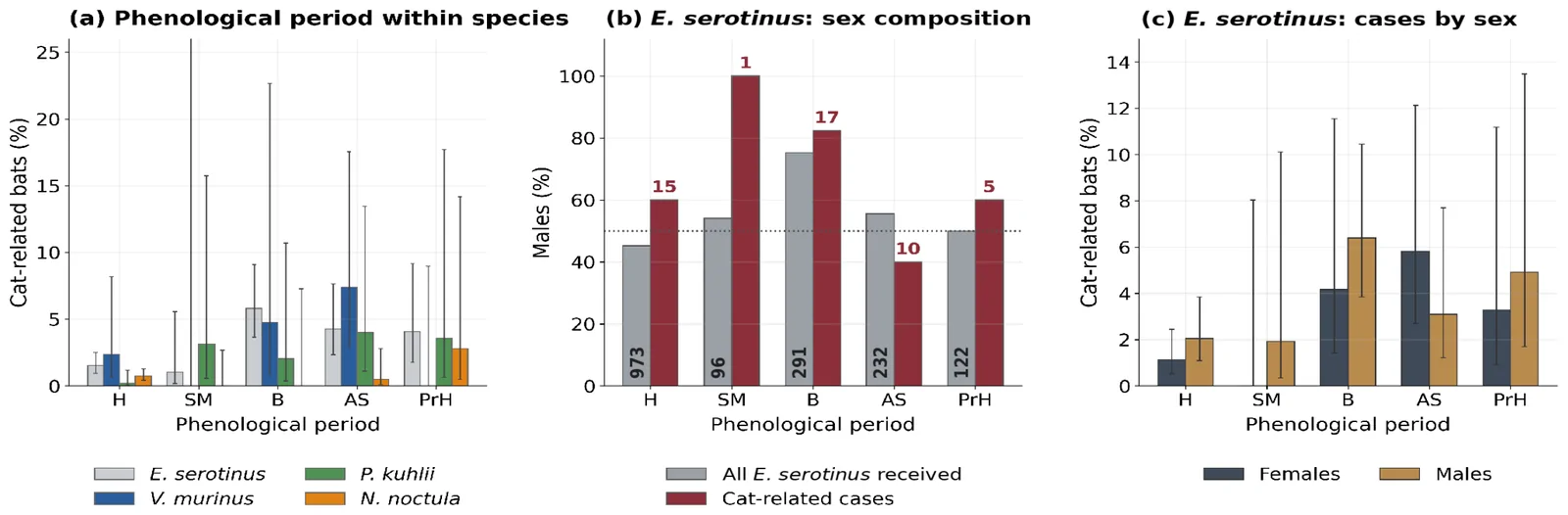
Phenological periods are abbreviated as H, hibernation (November–March); SM, spring migration (April); B, breeding (May–July); AS, autumn swarming (August–September); and PrH, pre-hibernation fat accumulation (October). (**a**) Percentage of bats received for which a cat was recorded, by period and species, with Wilson 95% confidence intervals. The upper confidence limit for *V. murinus* in the spring migration period (43.45%; 0 of 5 bats) lies beyond the vertical axis. (**b**) Percentage of males among the *E. serotinus* received and among the cat-related cases, by period; the number of individuals received is given inside each grey bar and the number of cases above each red bar; the dotted line marks parity. (**c**) Percentage of cat-related records among female and male *E. serotinus*.

Cat-related cases were more frequent in the activity season (April to October) than in hibernation (November to March) in *E. serotinus* (OR = 2.98, Fisher’s exact test, *p* < 0.001) and in *P. kuhlii* (OR = 15.55, *p* = 0.004), but not in *V. murinus* (*p* = 0.702) or *N. noctula* (*p* = 0.748) (Table 4, Figure 3a). The estimate stratified by species was 2.58 (95% CI 1.58–4.21), and the test of homogeneity gave *p* = 0.054. The percentage of cat-related cases was highest in the breeding period in *E. serotinus* (17 of 293, 5.8%) and in the autumn swarming period in *V. murinus* (4 of 54, 7.4%) and *P. kuhlii* (2 of 50, 4.0%); in *N. noctula*, no cases occurred among the 49 bats received in the breeding period or the 140 received in the spring migration period (Table S3a).

Males were over-represented among the cases in three of the four species, and the estimate stratified by species was 1.97 (1.20–3.23, *p* = 0.006), homogeneous between species (*p* = 0.223; Table 4). The excess reached significance only in *P. kuhlii* (*p* = 0.035), in which all six cases were male. In *E. serotinus*, the only species with cases in nine of the ten period-by-sex cells, the male excess among cases followed the sex composition of the bats received (Figure 3b, Table S3c). In the breeding period, males made up 75.3% of the *E. serotinus* received and 82.4% of the cases, and the percentage of cat-related cases was 4.17% among females and 6.36% among males (OR = 1.56, 0.44–5.60, *p* = 0.77); across the whole activity season it was 3.93% among females and 4.76% among males (OR = 1.22, 0.58–2.56, *p* = 0.71; Figure 3c).

Adults and first-year bats carried a cat-related record with the same frequency in all four species; the stratified estimate was 0.98 (0.60–1.62, *p* = 0.95), and the species did not differ in this respect (*p* = 0.998; Table 4).

### 3.4. Place of the Find

The place of the find was determined for 42 of the 75 cat-related cases of the four species (56%) and for 1805 of the remaining 4603 bats (39%) (Table 5). Cat-related cases were found most often outdoors, on the ground or in the street (20 of the 42), indoors in dwellings and offices (11) and on balconies or window ledges (6); three were found in the yards of private houses. Only two cases occurred in a stairwell or building entrance and none in a basement or attic, although these communal spaces accounted for 17.2% and 3.3% of the other records with a recorded place. The floor of the building was recorded for 12 cases and 1197 other records; the median floor was the fifth in both groups.

At the Derzhprom building, where the type of place was recorded for 990 of the 1099 bats of the winter of 2014/15, every case with a recorded place was inside the building: 92 in open indoor spaces, where 11.4% (95% CI 9.4–13.8) of the bats received were cat-related, and 13 in the basement, where the figure was 16.2% (9.7–25.8); no case occurred in a closed room (0 of 68), in a passage between building sections (0 of 33) or outdoors (0 of 5) (Table 5). No statistical comparison between places was made (Note S1).

### 3.5. Outcome of the Cat-Related Cases and the Injuries Recorded

**Figure 4:**
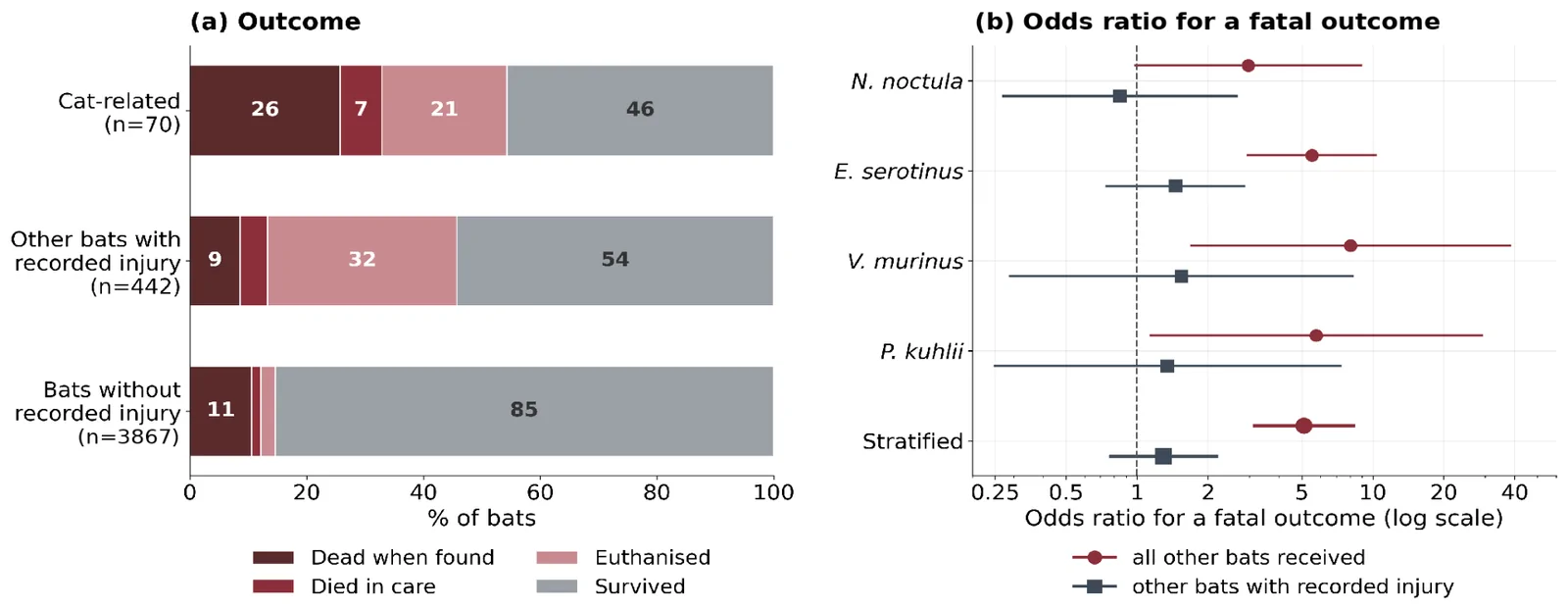
A fatal outcome comprises bats dead when received, bats that died in care and bats that were euthanised. (**a**) Outcome of the bats received, by group. (**b**) Odds ratio for a fatal outcome in cat-related cases, by species and stratified by species, against all other bats received (circles) and against other bats in whose record an injury was described (squares), on a logarithmic scale.

The outcome was determined for 4379 of the 4678 bats of the four species (93.6%) and for 70 of the 75 cat-related cases. Of these 70, 38 ended fatally (54.3%, 95% CI 42.7–65.4), 52 (74.3%, 63.0–83.1) reached the centre alive, and 32 (45.7%, 34.6–57.3) were released. Cat-related cases were more often dead on receipt and more often euthanised, and correspondingly less often released, than the other bats received (Figure 4a).

Compared with all other bats received, cat-related cases were about five times more likely to end fatally, and the species did not differ in this respect (stratified estimate 5.10, 95% CI 3.14–8.26, *p* < 0.001; homogeneity *p* = 0.710; Table 6, Figure 4b). Compared with the 442 other bats in whose record an injury was described, of which 45.7% ended fatally, cat-related cases were not more likely to end fatally in any species; the stratified estimate was 1.30 (0.77–2.17, *p* = 0.33), again homogeneous between species (*p* = 0.873). Of the 3867 bats without a recorded injury, 14.6% ended fatally. Cat-related cases made up 70 of the 512 bats with any recorded injury (13.7%, 11.0–16.9).

At least one injury was described in the note of 49 of the 77 cat-related cases of the general dataset (Table 7). Wounds on the head, trunk or limbs were recorded in 18 cases, fractures of the wing bones in 13, holes or tears of the wing membrane in 13, haematomas, swelling or bleeding in 13, and the loss of a foot, a wing or part of the body in 5. The outcome differed by type of injury: 11 of the 13 bats with a fracture and all 5 with a lost body part died, compared with 3 of the 13 with damage to the wing membrane.

### 3.6. The Derzhprom Building: The Winter of 2014/15 and the Years After

Of the 163 bats classified as killed by cats in the winter of 2014/15, 162 were *N. noctula*, and one was *E. serotinus*. No cause of death was assigned to 846 of the remaining 936 bats; the other 90 had died of debilitation, from contact with oil products or other chemicals, or had been killed by people. Sex was determined for 46 of the 163 cases; 25 of them (54.3%) were male, against 58.1% males among all bats received from the building, a difference that was not significant. Cases were recorded from September 2014 onwards and accumulated through the winter, with 114 of the 163 in March 2015 alone.

Over the rest of the period, from January 2013 to December 2024 excluding the winter of 2014/15, 18 cases were identified among the 3805 bats received from the building (0.47%, 95% CI 0.30–0.75), all of them *N. noctula*: two in 2013, one in 2014, nine in 2016, five in 2017 and one in 2018, and none thereafter (Figure 1a). The percentage of bats received from the building as remains, that is, as wings, heads, jaws or forearms rather than as whole animals, followed the same course (Table S5). It was 0.3% and 1.2% in the two winters before the event, rose to 11.4% (9.6–13.5) in 2014/15, fell to 1.0% in 2015/16 and to 0.4% in 2016/17, and no bats have been received as remains in any winter since. The winter of 2014/15 differed from the two that preceded it (Fisher’s exact test, OR = 14.4, *p* < 0.001) and from all that followed (OR = 42.6, *p* < 0.001).

Two changes at the building fell within this period. The free-roaming cats were killed by an unidentified person in the summer of 2015, and in the summer of 2016 most of the crevices opening into the building were sealed with polyurethane foam during repair work [19]. Over the same period, the number of bats received from the building in winter fell from 1051 in 2014/15 to 281 in 2016/17. At the main building of the V.N. Karazin Kharkiv National University, 200 m from Derzhprom, 3354 bats were received over the same twelve years, 94% of them *N. noctula*, and none carried a cat-related record (0 of 3354; upper 95% bound 0.11%).

## 4. Discussion

### 4.1. Two Regimes of Cat Predation on Urban Bats

This study provides the first quantitative assessment of cat predation on bats in Eastern Europe, and the dataset is, to our knowledge, the largest of its kind. Most of the global evidence consists of single observations made without any measure of how many bats were available to be taken [13]; records of rehabilitation centres, despite their known biases [21,34], are among the few sources from which such a rate can be derived. The value of the material lies less in the headline figure, 1.59% of the bats received in finds of four or fewer, than in what it reveals about the structure of the phenomenon. Cat predation on urban bats operated in two regimes that differ in mechanism, in scale and in the measures that can address them, and pooling them would obscure both.

The first regime is a dispersed background of attacks on individual bats at a stable low rate. No second site comparable to Derzhprom emerged in twelve years across 258 settlements; the annual percentage showed no trend and did not vary with the number of bats received from a settlement. The higher rate in Kharkiv narrowed as larger finds were excluded and was not significant among finds of a single bat, so it reflects how bats reach the centre in a large city rather than a concentration of predation. That almost every case was a find of a single bat follows from the same mechanism: a cat takes one bat at a time, and a resident reports one encounter, whereas finds of several bats come from rescue operations inside buildings, from which cats are absent. The cases were not concentrated in any type of location other than by their absence from the communal interior spaces of buildings. This matches the hunting behaviour of owned cats, which take bats only incidentally [11], and the Italian rehabilitation data, in which predation was attributed to owned cats with access to terraces and gardens and no event on the scale of Derzhprom was recorded [21].

The proportion of admissions attributable to cats in Italy, 28.7% of adult bats [21], is an order of magnitude above our 1.59%. The difference is more plausibly one in how bats reach the centre than in predation pressure: in Kharkiv most single-bat finds are *N. noctula* that entered a building in winter and are uninjured, whereas in Italy a bat reaches a centre mainly because something has happened to it. When the comparison is restricted to injured bats, the share attributable to cats in our material, 13.7%, is of the same order as the 45.5% of traumatic deaths attributed to cats in German post-mortem material [39], allowing for the different populations from which the two figures are calculated.

The second regime is roost-scale predation, which arises where cats gain access to an aggregation of bats flying within an enclosed space. Bats wintering in the Derzhprom building emerged periodically into corridors and staircases, where cats had unrestricted access and where flight offered neither height nor cover [19]. That the cats had specialised on this resource is indicated by the higher body mass of the bats they killed relative to healthy and debilitated individuals in the same sample [19], and by the concentration of cases in the basement and other internal spaces, with none recorded outdoors or in closed rooms. Comparable events have been recorded wherever that configuration recurs: repeated predation at a discovered roost, in some cases to the point of extirpation, is a recognised pattern in owned cats [21]; at a cave roost, cats specialised on bats, took them independently of species abundance and appeared to pass the preference to kittens [24]; a single cat killed at least 102 *Mystacina tuberculata* Gray, 1843, at roost trees within seven days [40]; and bat remains occurred in 30% of cat faeces at an urban horseshoe bat roost [41]. Whether the structure is a cave, a tree, an attic or a building appears to be secondary.

The sequence of events at Derzhprom suggests which element of that configuration is decisive. The proportion of bats received as remains fell tenfold after the cats were killed in 2015 and to zero after the crevices were sealed in 2016, and no cat-related record has been made since 2018. Neither change was undertaken for the bats, and both coincided with a decline in the number of bats trapped inside the building, so the effects cannot be fully separated. The pattern is nonetheless consistent with access, rather than the presence of cats, being the limiting factor: removal of cats is neither permanent nor, on this evidence, sufficient, whereas closing access ended the predation. Where such traps arise cannot be predicted from the type of building, since the main building of Karazin University, 200 m away and comparable in size and in its hibernating aggregation of *N. noctula*, yielded no case in 3354 records; the appropriate response is monitoring of large winter aggregations rather than prediction.

### 4.2. Which Bats Are Taken: Availability Rather than Selection

The differences between species reflect availability rather than selection. We do not suggest that cats choose particular species: a cat takes the bat it can reach, and which species that is follows from where and when each species roosts and flies. The species most affected were those that roost in buildings and forage low. *Eptesicus serotinus* and *V. murinus* were four to five times more likely than *N. noctula* to carry a cat-related record over the year, and in the activity season, in which all four species are present, *P. kuhlii* joined them with odds seven to ten times higher. *Nyctalus noctula* forages high above the built-up area, occupies elevated crevices and is present in the city mainly in winter. Derzhprom shows that it is flight height rather than hibernation as such that keeps it beyond reach: once it flies within an enclosed space, it becomes available.

This corresponds to the global pattern, in which the species taken are those roosting close to people and hunting near the ground [13], to the Italian data, in which building-roosting species made up 80% of victims [21], and to the German post-mortem series, where cat predation affected chiefly *Pipistrellus* spp., *Plecotus auritus* (Linnaeus, 1758), *V. murinus* and *Myotis mystacinus* (Kuhl, 1817), while trauma in *N. noctula* arose from confinement in structures and the destruction of tree roosts rather than from cats [42]. The species taken differ between Italy (*P. kuhlii*, *H. savii*) and Kharkiv (*E. serotinus*), but all of them roost in buildings and remain in settlements throughout the year, whereas *N. noctula* occupies buildings mainly during hibernation. *Pipistrellus kuhlii* shows how an annual figure can conceal this: three-quarters of the *P. kuhlii* were received during hibernation, when 1 of 480 carried a cat-related record, against 5 of 159 in the activity season.

The excess of males among the cat-related cases of *E. serotinus* likewise reflects the composition of the bats available rather than a difference in vulnerability: in the breeding period, when most cases occur, males make up three-quarters of the *E. serotinus* received and a similar share of the cases, and the per capita frequency does not differ between the sexes. Female *E. serotinus* leave the urban core for the breeding season [43], in keeping with the sexual segregation of temperate bats in summer [44] and the greater habitat selectivity of females in urban landscapes [45]. The Italian finding that females were taken more often [21] is the mirror image, since the species affected there breed in buildings, and the male bias in the German material, 47 of 65 sexed victims [39], is close to ours. Age had no detectable effect in any species, as in Italy [21]. The composition of victims by sex or age cannot be read as vulnerability until it has been set against the composition of admissions in the same period.

### 4.3. Mortality and Injuries: The Frequency of Attacks Rather than the Severity of Injury

Cat attacks were fatal for more than half of the bats concerned, but not detectably more often than injuries of other origin: against other bats received with a recorded injury, the odds of a fatal outcome were not raised, and the estimate was homogeneous across species. The two comparison groups bracket the appropriate one, because the free text carries less detail for other bats than for cat-related cases and a lighter injury may go unrecorded; the odds ratio for a fatal outcome therefore lies between 1.30 and 5.10, and the lower, conservative value does not differ from unity. The burden imposed by cats therefore arises from the frequency of attacks rather than from the severity of any one of them. The injuries recorded were those expected from a cat’s teeth and claws, and the outcome followed their type: fractures and the loss of a body part were almost always fatal, whereas all bats whose only recorded injury was damage to the wing membrane survived. This is consistent with post-mortem evidence from Germany, where cat predation accounted for almost half of all traumatic deaths and was the most frequent single identified cause of death [39], and where the proportion of cat-killed bats that had been euthanised, about a third, is comparable to ours.

Post-mortem data also reveal a consequence not visible in rehabilitation records. Cat predation was the strongest predictor of bacterial infection in the German series, with almost half of the cat-killed bats carrying bacterial disease, predominantly *Pasteurella multocida* transmitted by the bite [39,42]. Bats that survive the attack itself thus remain at risk of septicaemia, pneumonia or abscess formation in the following days, which may account for the share of cat-related cases in our material that died in care or were euthanised after admission.

Our attribution is likely to be conservative. Forensic analysis has recovered cat DNA from two-thirds of bat wounds submitted by rehabilitators as injuries of unknown origin [23], so part of our comparison group will also have been injured by cats, and this acts against detecting a difference.

### 4.4. Cats at the Interface Between Reservoir Hosts and People

*Eptesicus serotinus*, the species most often taken by cats in our material, is one of the principal reservoir species of European bat lyssavirus type 1 (EBLV-1). Six lyssavirus species circulate in European bats; classical rabies virus is not among them [26,27]. Transmission across the cat–bat interface is documented: two domestic cats in France were infected with EBLV-1, both had bitten people, and in one case the carcass of an *E. serotinus* carrying a closely related strain was recovered in the same locality two months later [28]. West Caucasian bat virus, which lies outside the phylogroup of classical rabies virus and against which current vaccines and immunoglobulins are therefore unlikely to protect [30,31], caused fatal rabies in a domestic cat in Italy in 2020, with a nearby colony of *Miniopterus schreibersii* (Kuhl, 1817) as the probable source [27,29]. In Ukraine, EBLV-1 has been detected in *E. serotinus*: six of 85 individuals tested were positive, three from Kyiv and three from Kharkiv, while none of 21 *N. noctula* were positive [46]. This remains the only systematic screening of bats for lyssaviruses in the country, although lyssavirus infection has been recorded in a Ukrainian cat that had been in contact with a bat [32]. In Canada, cats delivered four to five times as many bats for rabies testing as dogs and, although those bats were less often positive, accounted for the greater share of human exposure [47]. The same interface has been described as a neglected rabies risk in Ecuador [48] and identified as a One Health problem requiring systematic data [25].

The present study supplies one such measure. Among the four species in finds of four bats or fewer, 52 of the 70 cat-related cases with a determined outcome reached the centre alive, that is, three-quarters, and 32 were released. The contact is therefore frequent, and it can be quantified from rehabilitation records.

### 4.5. Limitations

The percentages reported here are lower bounds, since a cat is recorded only when the finder mentions one or when the injuries are attributed to one. Rehabilitation records describe the intersection between bats and people rather than a population [34], a limitation shared by most of the global evidence [13]. Three-quarters of the bats in finds of four or fewer derive from a single large city, and the estimates should be read as applying to Kharkiv. Attribution rests on the finder’s account and the pattern of injury, without laboratory confirmation. Predation by cats has been reported to be more frequent in rural and low-density housing, where cats are more often allowed out at night [21]; the present data cannot test this, because no settlement other than Kharkiv contributed more than one case and settlement size is confounded with sample size (Note S2). The record of the place of the find is incomplete and unevenly so, and no quantitative comparison between places is therefore attempted (Note S1). The material is a working database of a rescue centre rather than a standardised survey, and the screening of its free text should be read as a conservative filter on it.

## 5. Conclusions

Cat predation on bats in the urban landscape of Eastern Europe operates in two regimes that should not be pooled. Dispersed attacks on individual bats occurred at a stable rate, close to 1.6% of the bats received singly or in small numbers, without trend over twelve years and without a second cluster anywhere in the country. Roost-scale events, exemplified by the Derzhprom building, arose where cats gained continuous access to an aggregation of bats flying within an enclosed space, and ended when that access was closed. Which bats are taken follows from where they roost and fly rather than from any choice by the cat: building-roosting species were taken most often, and the sex and age composition of the victims reflected the composition of the bats available. Cat attacks were fatal for more than half of the bats concerned, but not more often than injuries of other origin, so the burden arises from the frequency of attacks. Because the species most often taken is also a principal reservoir of European bat lyssavirus type 1, and because three-quarters of the bats taken by cats reach people alive, the contact quantified here is relevant to public health as well as to conservation.

## Figures and Tables

**Table 1:** Subsets of the material used in this study (Ukraine, 2013–2024), the cases identified in each, and the sections in which they are used.

Subset	Finds	Bats	Cat-Related Cases	Identification of Cases	Used in
All records, January 2013 to December 2024	—	41,910	258	combined, see below	—
General dataset (all sites except Derzhprom)	4583	37,006	77	text screening	Section 3.1
Finds of ≤15 bats	4310	6461	77	text screening	Section 3.1, Note S2
Finds of ≤4 bats (main threshold)	4114	4770	76	text screening	Section 3.1, Section 3.2, Section 3.3, Section 3.4 and Section 3.5
Four modelled species	—	4678	75	text screening	Section 3.2, Section 3.3, Section 3.4 and Section 3.5
Settlement recorded	—	4761	76	text screening	Section 3.1
Outcome determined	—	4379	70	text screening	Section 3.5
Place of find determined	—	1847	42	text screening	Section 3.4
Derzhprom building, January 2013 to December 2024	—	4904	181	163 from specimens + 18 by screening	Section 3.6
Winter 2014/15 (September 2014 to March 2015)	—	1099	163	cause of death from the specimen [19]	Section 3.4 and Section 3.6
Other seasons	—	3805	18	text screening	Section 3.6

A find is all bats received from one address on one date. The subsets restricted to the four modelled species, and to bats with a recorded settlement, outcome or place of find, are subsets of the finds of four bats or fewer, whose definition is given in Section 2.3. At the Derzhprom building, the 163 cases of the winter of 2014/15 were classified from the specimens, and the 18 cases of the other seasons were classified by screening the record text, as for the general dataset.

**Table 2:** Cat-related cases in the two datasets, 2013–2024.

	Finds	Bats	Cat-Related Cases	% of Bats	95% CI
General dataset					
finds of 1 bat	3681	3681	75	2.04	1.63–2.55
finds of 2–4 bats	433	1089	1	0.09	0.02–0.52
finds of 5–9 bats	131	858	0	0.00	0.00–0.45
finds of 10–19 bats	99	1426	1	0.07	0.01–0.40
finds of 20–49 bats	98	3210	0	0.00	0.00–0.12
finds of 50–199 bats	98	9851	0	0.00	0.00–0.04
finds of ≥200 bats	43	16,891	0	0.00	0.00–0.02
all finds	4583	37,006	77	0.21	0.17–0.26
finds of ≤4 bats	4114	4770	76	1.59	1.27–1.99
finds of ≤15 bats	4310	6461	77	1.19	0.95–1.49
Derzhprom building					
winter 2014/15 only	—	1099	163	14.83	12.87–17.03
all other seasons	—	3805	18	0.47	0.30–0.75

A find is all bats received from the same address on the same date. Confidence intervals are Wilson intervals for a binomial proportion. The row for finds of up to 15 bats is not the sum of the rows above it, because the class of 10–19 bats is divided by the threshold of 15.

**Table 3:** Cat-related cases by species among bats received in finds of four or fewer individuals, general dataset, 2013–2024.

Species	Bats Received	Cat-Related Cases	% of Bats (95% CI)	Odds Ratio (95% CI)	*p*
*Nyctalus noctula*	2069	14	0.68 (0.40–1.13)	reference	—
*Eptesicus serotinus*	1755	48	2.74 (2.07–3.61)	4.13 (2.27–7.51)	<0.001
*Vespertilio murinus*	206	7	3.40 (1.66–6.85)	5.16 (2.06–12.94)	<0.001
*Pipistrellus kuhlii*	648	6	0.93 (0.43–2.01)	1.37 (0.53–3.58)	0.52
*Other species combined*	92	1	1.09 (0.19–5.87)	not modelled	—

Odds ratios are from a binomial generalised linear model with species as the only predictor and *N. noctula* as the reference level. Percentages are given with Wilson 95% confidence intervals. Species represented by fewer than 100 individuals were combined and excluded from the model.

**Table 4:** Odds ratios for phenological period, sex and age within each species, general dataset, finds of four bats or fewer.

Species	Activity Season vs. Hibernation	Breeding Period vs. Rest of Year	Males vs. Females	Adults vs. First-Year
*Nyctalus noctula*	0.64 (0.14–2.89)	1.39 (0.08–23.67)	3.09 (0.86–11.12)	1.02 (0.30–3.49)
*Eptesicus serotinus*	2.98 (1.61–5.53)	2.80 (1.53–5.13)	1.67 (0.92–3.03)	0.99 (0.53–1.84)
*Vespertilio murinus*	1.82 (0.34–9.61)	1.48 (0.17–12.88)	0.90 (0.20–4.14)	0.85 (0.18–3.91)
*Pipistrellus kuhlii*	15.55 (1.80–134.14)	2.44 (0.28–21.29)	10.88 (0.61–193.97)	1.04 (0.17–6.30)
Stratified estimate	2.58 (1.58–4.21)	2.50 (1.44–4.36)	1.97 (1.20–3.23)	0.98 (0.60–1.62)
Test of homogeneity	*p* = 0.054	*p* = 0.747	*p* = 0.223	*p* = 0.998

Values are odds ratios with 95% confidence intervals from the corresponding 2 × 2 tables. Stratified estimates are Mantel–Haenszel estimates with species as the stratum; the test of homogeneity is the Breslow–Day–Tarone test. The activity season is April to October and hibernation November to March; the breeding period is May to July. Age compares adults with first-year bats and excludes non-volant juveniles. Full calculations are given in Table S3b.

**Table 5:** Place of the find: the composition of cat-related cases and of other records in the general dataset, and the frequency of cat-related cases by type of place at the Derzhprom building in the winter of 2014/15.

**General Dataset, Records for Which a Place Was Determined**					
**Place of the Find**	**Cat-Related Cases, n**	**%**	**95% CI**	**Other Records, n**	**%**
outdoors, on the ground or in the street	20	47.6	33.4–62.3	648	35.9
indoors	11	26.2	15.3–41.1	373	20.7
balcony or window	6	14.3	6.7–27.8	377	20.9
yard of a private house	3	7.1	2.5–19.0	37	2.0
stairwell or building entrance	2	4.8	1.3–15.8	310	17.2
basement or attic	0	0.0	0.0–8.4	60	3.3
Total	42			1805	
**Derzhprom building, winter 2014/15, all bats received**					
**Type of place**	**Bats**	**Cat-related cases**		**%**	**95% CI**
open indoor space	804	92		11.4	9.4–13.8
basement	80	13		16.2	9.7–25.8
closed room	68	0		0.0	0.0–5.3
passage between building sections	33	0		0.0	0.0–10.4
outdoors	5	0		0.0	0.0–43.4
not specified	109	58		53.2	43.9–62.3
Total	1099	163		14.8	

The two panels are built differently, and their percentages are not comparable. In the general dataset, the place was recovered from the free text and is known for 42 of the 75 cat-related cases but only 1805 of the 4603 other records; the percentages there are the composition of each group, and each percentage column sums to 100. At the Derzhprom building, the type of place is a field of the dedicated database, recorded for 990 of the 1099 bats, and the percentage is the proportion of the bats in each place for which a cat was recorded. Confidence intervals are Wilson intervals for a binomial proportion. No statistical comparison between places is made, for the reasons set out in Note S1.

**Table 6:** Fatal outcome by species and group, general dataset, finds of four bats or fewer.

Species	Cat-Related	Other Bats with Recorded Injury	Bats Without Recorded Injury	OR vs. All Other Bats	OR vs. Injured Bats
*Nyctalus noctula*	5/14 (35.7)	63/159 (39.6)	247/1803 (13.7)	2.96 (0.99–8.89)	0.85 (0.27–2.64)
*Eptesicus serotinus*	26/43 (60.5)	103/201 (51.2)	242/1389 (17.4)	5.52 (2.96–10.29)	1.46 (0.74–2.85)
*Vespertilio murinus*	4/7 (57.1)	13/28 (46.4)	12/148 (8.1)	8.05 (1.70–38.16)	1.54 (0.29–8.18)
*Pipistrellus kuhlii*	3/6 (50.0)	23/54 (42.6)	63/527 (12.0)	5.76 (1.14–28.99)	1.35 (0.25–7.30)
All species, stratified	38/70 (54.3)	202/442 (45.7)	564/3867 (14.6)	5.10 (3.14–8.26)	1.30 (0.77–2.17)
Test of homogeneity				*p* = 0.710	*p* = 0.873

Values in the first three columns are the number of bats with a fatal outcome out of the number of bats with a known outcome, with the percentage in brackets. A fatal outcome comprises bats dead when found, bats that died in care and bats that were euthanised. Odds ratios are from the corresponding 2 × 2 tables with 95% confidence intervals; stratified estimates are Mantel–Haenszel estimates with species as the stratum and the test of homogeneity is the Breslow–Day–Tarone test.

**Table 7:** Injuries described in the free-text notes of the 77 cat-related cases of the general dataset, 2013–2024.

Injury Recorded	Cases, n	% of Cases	Fatal Outcome, n
wounds on the head, trunk or limbs	18	23.4	10
fracture of the wing bones	13	16.9	11
damage to the wing membrane	13	16.9	3
haematoma, swelling or bleeding	13	16.9	9
loss of a body part	5	6.5	5
any injury described	49	63.6	29
no injury described	28	36.4	10
Total	77		39

The categories are not mutually exclusive, so the rows do not sum to the total. Five of the 18 wounds were recorded only as “bitten by a cat”. A fatal outcome comprises bats dead when found, bats that died in care and bats that were euthanised. The category assigned to each case is given in Table S1.

## Data Availability

The occurrence records underlying this study are publicly available through the Global Biodiversity Information Facility (https://doi.org/10.15468/7t4zgc and https://doi.org/10.15468/8xfwhc). All cat-related records, with the original and translated free-text notes, are given in Table S1. The full output of the statistical analyses is given in Tables S2–S5 and Notes S1–S3.

## Supplementary Materials

The following supporting information can be downloaded at: https://www.mdpi.com/article/10.3390/ani16213536/s1, Table S1: Cat-related records of the general dataset and of the Derzhprom building, one row per individual, with locality, coordinates, species, sex, age, outcome, the category of injury recorded and the original and translated free-text notes; Table S2: Full output of the species model at each threshold on the size of the find, with Wald, likelihood-ratio and exact confidence intervals and tests; Table S3: Percentages by phenological period and species, odds ratios within species with stratified estimates and tests of homogeneity, and the sex composition and per capita frequency of *Eptesicus serotinus* by period; Table S4: Species and phenological composition of finds of one and of two to four bats; Table S5: Bats received from the Derzhprom building as remains, by winter season; Note S1: The place of the find, and the reasons for making no statistical comparison between categories of place; Note S2: The effect of settlement size on the percentage of cat-related records; Note S3: The effect of the threshold on the size of the find on the odds ratios between species and on the difference between Kharkiv and the rest of the country.
