# Supplementary material for: Cat Predation on Bats in an Urban Landscape of Eastern Europe: Twelve Years of Rescue-Centre Records
Source: Animals (Basel). 2026 Nov 9;16(21):3536. doi: 10.3390/ani16213536 (PMC13762370; doi:10.3390/ani16213536)
Supplement: Supplementary file 1 [file animals-16-03536-s001.zip › Vlaschenko_et_al_Cat_bats_UBRC_Supplementary_20261024 - edited.pdf]

Supplementary Materials

Cat predation on bats in an urban landscape of Eastern Europe: twelve years of rescue-centre records

Anton Vlaschenko[1,2,3,4,†,*], **Maryna Yerofeieva**[1,5,†], **Alona Shulenko**[1,5], **Valeriia Bohodist**[1,2,6], **Kateryna Zemliana**[1,5], **Andrii Kulynych**[1,2], **Veronika Putiatina**[1,5] and **Alona Prylutska**[1,7]

Table S1 is provided as a separate Excel file (Table_S1_cat_records_UBRC_20261024.xlsx) and is not reproduced here. It lists every cat-related case individually, with locality, coordinates, species, sex, age, outcome, the category of injury described in the note (column injuryCategory; Section 2.5 of the main text) and the original free-text note with its English translation.

Note S1. The place of the find

No statistical comparison between categories of place is reported, for three reasons. In the general dataset the composition of places depends on how much detail a record carries: among records without a cat, short entries give 30.8% of finds outdoors and long entries 46.0%. Cat-related records are almost all detailed, only four of the 42 carrying a short entry, so a comparison between them and the remaining records would partly reproduce the difference between detailed and undetailed records. Stratifying by the length of the entry does not resolve this, because one stratum is then almost empty. At the Derzhprom building the incompleteness runs the other way: among the 109 bats for which no type of place was recorded, 53.2% were cat-related, compared to 11.4% in open indoor spaces, since a type of place was not entered for scattered remains. The two datasets were also classified by different schemes and cannot be compared formally. The contrast reported in Section 3.4 is therefore qualitative, and the Wilson intervals given for categories in which no case was recorded state the upper bound consistent with the number of records available. The dedicated database records a more detailed position for part of the Derzhprom cases of the winter of 2014/15: staircases and stair landings (26), electrical boxes (17), the basement (10), and the porter's desk and lobby (8); 61 of the 105 cases have a specific recorded type of place overall; the remaining 44 carry only the broader type of place.

Note S2. The effect of settlement size

Among finds of four bats or fewer, a place of origin is recorded for 4,761 of the 4,770 individual bats; the remaining nine cannot be assigned to any place. These 4,761 bats come from 253 of the 258 places represented in the whole material, with the five places not represented here having sent bats to the centre only in finds of more than four individuals. Kharkiv contributed 3,590 of the 4,761 bats (75.4%) and 65 of the 76 cat-related cases; in the whole material, which includes finds of more than four individuals, the share of Kharkiv is 60.9%. The places after Kharkiv each contributed far fewer bats: Zaporizhzhia 107, Dnipro 84 and Kyiv 68, while 207 of the 253 places contributed fewer than five bats each. Cat-related cases come from 12 of the 253 places; the remaining 241 places yielded no case, and outside Kharkiv each of the eleven places concerned yielded exactly one case.

The percentage of cat-related records was not modelled against the size of the settlement. No other settlement contributed more than one case, and the largest sample from any other settlement is 107 bats. Fitting the percentage against settlement population returns an odds ratio of 0.35 per tenfold increase (95% CI 0.17-0.70, $p = 0.003$), which would indicate a higher risk in small settlements. The association is an artefact: settlement population and sample size are correlated ($r = 0.73$ on a logarithmic scale), so that a single case in a sample of one, six or nine bats yields a percentage of 100, 17 or 11. Excluding settlements from which fewer than ten bats were received reverses the direction of the estimate and removes its significance (odds ratio 7.31, 0.60-89.28, $p = 0.119$), and no higher threshold gives a significant result in either direction.

Note S3. The effect of the threshold on the size of the find

Odds ratios between species. The direction of the differences between species held at every threshold on the size of the find, but the size of the odds ratios rose steeply as larger finds were admitted. For *E. serotinus* the odds ratio relative to *N. noctula* was 3.05 in finds of a single bat, 4.13 in finds of four bats or fewer, 6.76 in finds of up to 15 bats and 57.64 when all finds were included; the corresponding values for *V. murinus* were 3.75, 5.16, 8.52 and 74.74, and for *P. kuhlii* 1.21, 1.37, 1.85 and 4.20 (Table S2, Figure 2b of the main text). This rise does not mean that the difference between the species is greater when larger finds are counted. Finds of more than four individuals add 32,236 bats to the general dataset and only one cat-related case, and nine in ten of those bats are *N. noctula*, the species against which the others are compared. Its percentage of cat-related records is therefore driven towards zero while the percentages in *E. serotinus* and *V. murinus* barely move, and the ratio between them grows for that reason alone. For *P. kuhlii* the odds ratio, which does not differ from unity at the three lower thresholds, becomes significant only when all finds are included, for the same reason.

Kharkiv against the rest of the country. The difference between Kharkiv and the rest of the country falls from fivefold over all finds (0.32% against 0.07%, OR = 4.77, p < 0.001) to twofold in finds of four bats or fewer (1.80% against 0.95%, OR = 1.92, p = 0.043) and is not significant in finds of a single bat (2.22% against 1.37%, OR = 1.63, p = 0.157).

Table S2. Full output of the model of species and the occurrence of a cat-related record, fitted at each threshold on the size of the find.

Threshold	Species	Bats	Cases	% of bats	95% CI of %	OR	Wald 95% CI	Exact 95% CI	p Wald	p LR	p Fisher	Model LR χ^2 (df), p
Finds of 1 bat	*Nyctalus noctula*	1307	13	0.99	0.58–1.69	reference						19.05 (3), 2.68e-04
	Eptesicus serotinus	1612	48	2.98	2.25–3.93	3.05	1.65–5.66	1.62–6.17	3.91e-04	1.09e-04	1.38e-04	
	Vespertilio murinus	193	7	3.63	1.77–7.30	3.75	1.48–9.51	1.25–10.24	5.46e-03	1.07e-02	9.30e-03	
	Pipistrellus kuhlii	499	6	1.20	0.55–2.60	1.21	0.46–3.20	0.38–3.44	6.99e-01	7.03e-01	7.97e-01	
Finds of ≤4 bats	*Nyctalus noctula*	2069	14	0.68	0.40–1.13	reference						31.56 (3), 6.49e-07
	Eptesicus serotinus	1755	48	2.74	2.07–3.61	4.13	2.27–7.51	2.23–8.13	3.48e-06	3.18e-07	4.68e-07	
	Vespertilio murinus	206	7	3.40	1.66–6.85	5.16	2.06–12.94	1.74–13.84	4.63e-04	1.85e-03	1.74e-03	
	Pipistrellus kuhlii	648	6	0.93	0.43–2.01	1.37	0.53–3.58	0.43–3.82	5.19e-01	5.28e-01	5.97e-01	
Finds of ≤15 bats	*Nyctalus noctula*	3489	14	0.40	0.24–0.67	reference						56.85 (3), 2.76e-12
	Eptesicus serotinus	1847	49	2.65	2.01–3.49	6.76	3.72–12.28	3.66–13.30	3.40e-10	1.75e-12	2.23e-12	
	Vespertilio murinus	211	7	3.32	1.62–6.69	8.52	3.40–21.33	2.87–22.82	4.82e-06	9.77e-05	1.04e-04	
	Pipistrellus kuhlii	813	6	0.74	0.34–1.60	1.85	0.71–4.82	0.58–5.13	2.11e-01	2.32e-01	2.46e-01	
All finds	*Nyctalus noctula*	31256	14	0.04	0.03–0.08	reference						240.25 (3), 8.42e-52
	Eptesicus serotinus	1946	49	2.52	1.91–3.31	57.64	31.77–104.59	31.24–113.22	1.40e-40	1.76e-48	4.01e-48	
	Vespertilio murinus	216	7	3.24	1.58–6.54	74.74	29.86–187.07	25.22–200.01	3.07e-20	4.41e-11	6.97e-11	
	Pipistrellus kuhlii	3195	6	0.19	0.09–0.41	4.20	1.61–10.93	1.32–11.64	3.30e-03	8.95e-03	7.83e-03	

Threshold	Species	Bats	Cases	% of bats	95% CI of %	OR	Wald 95% CI	Exact 95% CI	p Wald	p LR	p Fisher	Model LR χ^2 (df), p
Finds of ≤4 bats, activity season (Apr–Oct)	*Nyctalus noctula*	423	2	0.47	0.13–1.71	reference						19.21 (3), 2.47e-04
	Eptesicus serotinus	749	33	4.41	3.15–6.12	9.70	2.32–40.64	2.46–83.77	1.88e-03	1.51e-05	4.39e-05	
	Vespertilio murinus	119	5	4.20	1.81–9.46	9.23	1.77–48.21	1.48–97.61	8.39e-03	4.92e-03	6.80e-03	
	Pipistrellus kuhlii	159	5	3.14	1.35–7.15	6.83	1.31–35.59	1.10–72.19	2.24e-02	1.46e-02	1.85e-02	

Response: whether a cat was recorded for the individual. Predictor: species, with *Nyctalus noctula* as the reference level; binomial model with a logit link. Wald confidence intervals are from the model standard errors; exact intervals are conditional intervals from the 2 × 2 table. The model likelihood-ratio test compares the model with the null model and is given once per threshold. Odds ratios computed directly from the 2 × 2 tables agree with the model coefficients, as expected for a saturated model. A generalised estimating equations model clustered by find gave odds ratios of 4.11, 5.15 and 1.37 for finds of four bats or fewer, against 4.13, 5.16 and 1.37 from the model. In the activity-season block, bats whose month of receipt was not recorded are excluded.

Table S3a. Percentage of bats received for which a cat was recorded by phenological period and species.

Species	Phenological period	Bats received	Cat-related cases	% of bats	95% CI
Nyctalus noctula	hibernation	1638	12	0.73	0.42–1.28
	spring migration	140	0	0.0	0.00–2.67
	breeding	49	0	0.0	0.00–7.27
	autumn swarming	198	1	0.51	0.09–2.80
	pre-hibernation	36	1	2.78	0.49–14.17
Eptesicus serotinus	hibernation	985	15	1.52	0.93–2.50
	spring migration	98	1	1.02	0.18–5.56
	breeding	293	17	5.8	3.65–9.09
	autumn swarming	235	10	4.26	2.33–7.65
	pre-hibernation	123	5	4.07	1.75–9.16
Vespertilio murinus	hibernation	85	2	2.35	0.65–8.18
	spring migration	5	0	0.0	0.00–43.45
	breeding	21	1	4.76	0.85–22.67
	autumn swarming	54	4	7.41	2.92–17.55
	pre-hibernation	39	0	0.0	0.00–8.97
Pipistrellus kuhlii	hibernation	480	1	0.21	0.04–1.17
	spring migration	32	1	3.12	0.55–15.74
	breeding	49	1	2.04	0.36–10.69

Species	Phenological period	Bats received	Cat-related cases	% of bats	95% CI
	autumn swarming	50	2	4.0	1.10–13.46
	pre-hibernation	28	1	3.57	0.63–17.71

Bats whose month of receipt was not recorded (8 *N. noctula*, 21 *E. serotinus*, 2 *V. murinus* and 9 *P. kuhlii*) are excluded, so the rows do not sum to the species totals of Table 3.

Phenological periods: hibernation, November–March; spring migration, April; breeding, May–July; autumn swarming, August–September; pre-hibernation, October. Confidence intervals are Wilson intervals for a binomial proportion.

Table S3b. Odds ratios within each species for four contrasts, with stratified estimates.

Contrast	Species	Cases/bats, group 1	% group 1	Cases/bats, group 2	% group 2	Odds ratio	95% CI	p
Activity season (Apr–Oct) vs hibernation (Nov–Mar)	*Nyctalus noctula*	2/423	0.47	12/1638	0.73	0.64	0.14–2.89	0.7477
	Eptesicus serotinus	33/749	4.41	15/985	1.52	2.98	1.61–5.53	0.0003
	Vespertilio murinus	5/119	4.20	2/85	2.35	1.82	0.34–9.61	0.7017
	Pipistrellus kuhlii	5/159	3.14	1/480	0.21	15.55	1.80–134.14	0.0044
	Stratified by species					2.58	1.58–4.21	9.83e-05
	Test of homogeneity							0.054
Breeding period (May–Jul) vs rest of year	Nyctalus noctula	0/49	0.00	14/2012	0.70	1.39	0.08–23.67	1.0000
	Eptesicus serotinus	17/293	5.80	31/1441	2.15	2.80	1.53–5.13	0.0014
	Vespertilio murinus	1/21	4.76	6/183	3.28	1.48	0.17–12.88	0.5382
	Pipistrellus kuhlii	1/49	2.04	5/590	0.85	2.44	0.28–21.29	0.3816
	Stratified by species					2.50	1.44–4.36	8.70e-04
	Test of homogeneity							0.747
Males vs females	*Nyctalus noctula*	11/1116	0.99	3/935	0.32	3.09	0.86–11.12	0.1036
	Eptesicus serotinus	31/913	3.40	17/823	2.07	1.67	0.92–3.03	0.1069
	Vespertilio murinus	3/92	3.26	4/111	3.60	0.90	0.20–4.14	1.0000
	Pipistrellus kuhlii	6/349	1.72	0/287	0.00	10.88	0.61–193.97	0.0350
	Stratified by species					1.97	1.20–3.23	6.29e-03
	Test of homogeneity							0.223

Contrast	Species	Cases/bats, group 1	% group 1	Cases/bats, group 2	% group 2	Odds ratio	95% CI	p
Adults vs first-year	*Nyctalus noctula*	4/681	0.59	7/1213	0.58	1.02	0.30–3.49	1.0000
	Eptesicus serotinus	24/909	2.64	18/676	2.66	0.99	0.53–1.84	1.0000
	Vespertilio murinus	3/86	3.49	4/98	4.08	0.85	0.18–3.91	1.0000
	Pipistrellus kuhlii	2/217	0.92	3/340	0.88	1.04	0.17–6.30	1.0000
	Stratified by species					0.98	0.60–1.62	9.47e-01
	Test of homogeneity							0.998

Group 1 is the first-named category of each contrast. Stratified estimates are Mantel–Haenszel estimates with species as the stratum; the test of homogeneity is the Breslow–Day–Tarone test. p-values within species are from Fisher's exact test.

Table S3c. *Eptesicus serotinus*: sex composition and per capita frequency by phenological period.

Period	Bats received	% males received	Cases	% males among cases	Cases/bats, females	% females	Cases/bats, males	% males	Odds ratio (M vs F)	95% CI	p
hibernation	973	45.2	15	60.0	6/533	1.13	9/440	2.05	1.83	0.65–5.19	0.300
spring migration	96	54.2	1	100.0	0/44	0.0	1/52	1.92	1.73	0.06–52.67	1.000
breeding	292	75.3	17	82.4	3/72	4.17	14/220	6.36	1.56	0.44–5.60	0.772
autumn swarming	232	55.6	10	40.0	6/103	5.83	4/129	3.1	0.52	0.14–1.88	0.346
pre-hibernation	122	50.0	5	60.0	2/61	3.28	3/61	4.92	1.53	0.25–9.47	1.000
ALL ACTIVITY (Apr–Oct)	742	62.3	33	66.7	11/280	3.93	22/462	4.76	1.22	0.58–2.56	0.714

The last row combines the four periods of the activity season. Bats whose sex was not determined are excluded, so the totals are smaller than those of Tables S3a and S3b by 12, 2, 1, 3 and 1 individuals in the five periods. p-values are from Fisher's exact test. The breakdown by period and sex is reported for *E. serotinus* only: with 48 cases, nine of the ten period-by-sex cells are occupied, whereas in the other three species four or fewer cells are occupied.

Table S4. Species and phenological composition of finds of a single bat and of finds of two to four bats, general dataset, 2013–2024.

	Finds of 1 bat	Finds of 2–4 bats	p
**Bats**	3,681 (100)	1,089 (100)	
N. noctula	1,307 (35.5)	762 (70.0)	
E. serotinus	1,612 (43.8)	143 (13.1)	
V. murinus	193 (5.2)	13 (1.2)	
P. kuhlii	499 (13.6)	149 (13.7)	
other species	70 (1.9)	22 (2.0)	< 0.001
hibernation	2,431 (66.7)	792 (73.1)	
spring migration	229 (6.3)	48 (4.4)	
breeding	350 (9.6)	90 (8.3)	
autumn swarming	423 (11.6)	134 (12.4)	
pre-hibernation	213 (5.8)	19 (1.8)	< 0.001

Percentages are column percentages; those for phenological period are of the bats whose month of receipt was recorded. p-values are from a chi-squared test of the whole column pair. The two classes differ in both respects: *N. noctula* makes up 35.5% of single-bat finds and 70.0% of finds of two to four bats. Finds of four bats or fewer are used as the main threshold because they are those a resident reports after a single encounter, not because their composition matches that of single-bat finds; the model was refitted to single-bat finds alone, with the same result (Table S2).

Table S5. Bats received from the Derzhprom building (Kharkiv, Ukraine) as remains by winter season.

Winter season	Bats received	Received as remains	%	95% CI
2012/13	289	1	0.3	0.1–1.9
2013/14	501	6	1.2	0.5–2.6
**2014/15**	**1,051**	**120**	**11.4**	**9.6–13.5**
2015/16	520	5	1.0	0.4–2.2
2016/17	281	1	0.4	0.1–2.0
2017/18	349	0	0.0	0.0–1.1
2018/19	369	0	0.0	0.0–1.0
2019/20	195	0	0.0	0.0–1.9
2021/22	45	0	0.0	0.0–7.9
2022/23	81	0	0.0	0.0–4.5
2024/25	148	0	0.0	0.0–2.5

A winter season runs from November to March. Seasons in which fewer than 40 bats were received are omitted. The seasons 2012/13 and 2024/25 are incomplete, comprising January–March 2013 and November–December 2024 respectively, since the material covers January 2013 to December 2024. The 1,051 bats of 2014/15 are those received from November to March; the 1,099 bats of the 2014/15 season (September–March) used in the main text (Table 1) also include those received in September and October 2014. Remains were identified from the free-text fields. Confidence intervals are Wilson intervals for a binomial proportion. The winter of 2014/15 differs from the two that preceded it (Fisher's exact test, OR = 14.4, $p < 0.001$) and from all that followed (OR = 42.6, $p < 0.001$); 2015/16 does not differ from 2016/17 ($p = 0.671$) but does differ from the later seasons taken together, in which no remains were received ($p = 0.003$).
